# Plasma Concentrations and Maternal-Umbilical Cord Plasma Ratios of the Six Most Prevalent Carotenoids across Five Groups of Birth Gestational Age

**DOI:** 10.3390/antiox10091409

**Published:** 2021-09-02

**Authors:** Chelsey McConnell, Melissa Thoene, Matthew Van Ormer, Jeremy D. Furtado, Zeljka Korade, Thiago C. Genaro-Mattos, Corrine Hanson, Ann Anderson-Berry

**Affiliations:** 1 Department of Pediatrics, University of Nebraska Medical Center, Omaha, NE 68198, USA; chelsey.mcconnell@unmc.edu (C.M.); matthew.vanormer@unmc.edu (M.V.O.); zeljka.korade@unmc.edu (Z.K.); alanders@unmc.edu (A.A.-B.); 2 Department of Nutrition, Harvard T.H. Chan School of Public Health, Boston, MA 02215, USA; jfurtado@hsph.harvard.edu; 3 Munroe Meyer Institute, University of Nebraska Medical Center, Omaha, NE 68198, USA; thiago.mattos@unmc.edu; 4 College of Allied Health Professions, University of Nebraska Medical Center, Omaha, NE 68198, USA; ckhanson@unmc.edu

**Keywords:** carotenoids, pregnancy, maternal:cord ratio, prematurity

## Abstract

Carotenoids are antioxidant nutrients with the potential to provide protection against oxidative stress. Plasma carotenoid concentrations are lower in newborn infants compared to their mothers; however, limited information is available regarding how concentrations differ by gestational age. The objective of this research is to assess maternal and umbilical cord plasma carotenoid concentrations and maternal-umbilical cord plasma ratios across five groups of birth gestational age. Mother-infant dyads were enrolled at delivery for collection of maternal and umbilical cord blood. Plasma carotenoids were analyzed by HPLC and LC-MS/MS. Birth gestational age was categorized into five groups, and the Kruskal–Wallis test compared carotenoid concentrations and maternal-umbilical cord plasma ratios between these groups. A *p*-value of < 0.05 was considered statistically significant. 370 mother-infant dyads were included, with most infants delivered at early term (20.3%) or term (64.6%). Though maternal plasma concentrations increased with birth gestational age, we observed less variability in umbilical cord plasma concentrations, thus the maternal-umbilical cord plasma ratio also increased with birth CGA groups for lutein + zeaxanthin (*p* = 0.008), β-cryptoxanthin (*p* = 0.027), α-carotene (*p* = 0.030); β-carotene approached significance (*p* = 0.056). Additional research is needed to determine if carotenoid concentrations were physiologic to varying gestational ages or if they were impacted by factors associated with preterm birth.

## 1. Introduction

Carotenoids are fat-soluble pigments found in plants, fungi, bacteria, and algae [1]. There are more than 600 different carotenoids found in nature, of which 50 have been identified in the United States (US) diet and are present predominantly in fruits and vegetables [1,2]. The most prevalent carotenoids consumed in the US are α-carotene, β-carotene, lycopene, lutein, zeaxanthin, and β-cryptoxanthin [2]. Of these, α-carotene, β-carotene, and β-cryptoxanthin can be converted to retinol in the body and are therefore referred to as provitamin A carotenoids [2]. The remainder (lycopene, lutein, and zeaxanthin) are not endogenously converted to retinol, so are consequently referred to as non-provitamin A carotenoids [2]. There are many factors that affect blood and tissue carotenoid concentrations in pregnant women, such as obesity, smoking, alcohol consumption, and dietary intake [3,4,5,6]. Current literature reports that carotenoid concentrations in newborns are highly correlated with concentrations of these nutrients in the mother, though concentrations in umbilical cord blood have been demonstrated to be several times lower than those in maternal blood [7,8,9]. Although carotenoids are beneficial for both adult and fetal health, there are no recommendations for dietary intake or reference ranges for plasma concentrations for these nutrients in this patient population. Therefore, a gap exists in our knowledge with regards to the variability of maternal and umbilical cord plasma carotenoid concentrations throughout gestation.

Carotenoids have anti-inflammatory and antioxidant properties and can protect against oxidative damage and inflammation [1,10]. Plasma or tissue concentrations of these antioxidant nutrients may impact perinatal health outcomes, as oxidative stress in pregnancy plays a role in the development of pathologies such as preeclampsia, intrauterine growth restriction, infant small for gestational age (SGA) size, spontaneous abortion, and preterm birth [11]. Previous studies have found decreased carotenoid concentrations in maternal plasma and placenta in women with preeclampsia [12]. Similarly, increased carotenoid concentrations in pregnant women at 24–28 weeks gestation had lower risk of having an SGA infant, indicating a potential role for these compounds in maternal and infant health [13]. In addition, carotenoids are vital to embryogenesis and play important roles in the development of the eyes, brain, and immune systems of fetuses while in utero [7,14]. Carotenoids also have been shown to affect cognitive health and performance beginning in infancy and continuing through adulthood [7].

There currently is little understanding of how maternal and umbilical cord plasma carotenoid concentrations may differ depending on the timing of delivery (preterm versus term). Factors that could impact concentrations include maternal blood lipid changes during pregnancy, changes in placental surface area, and maternal body fat accumulation during pregnancy. For example, maternal hyperlipidemia is common amongst all pregnancies and lipid levels increase as pregnancy progresses [15]. Women in their third trimester have low-density lipoprotein (LDL) levels 35% higher than non-pregnant women [15]. Carotenoids, which are fat-soluble, are transported in the blood by these lipoproteins and the presence of lipoprotein receptors in the placenta allows for their uptake and transfer [2,7,16]. Therefore, these increased lipoproteins circulating in maternal blood in late gestation can potentially allow for increased carotenoid transfer through the placenta and into the fetal bloodstream. Likewise, significant increases in surface area of the placenta from mid-gestation to term increases the functional capacity of the placenta and may result in higher carotenoid transfer from mother to fetus [17,18,19,20]. Lastly, carotenoids are able to be stored in adipose tissue and maternal body fat accumulates during early pregnancy but is not readily used until late gestation when maternal fat is broken down and transferred to the fetus [16]. Since carotenoids are fat-soluble, the concentration in neonates may be dependent upon their body fat stores which are directly dependent upon their birth weight and gestational age [21]. Though multiple factors may impact carotenoid concentrations, few studies have compared birth carotenoid concentrations in mother-infant dyads at varying gestational ages. However, preterm infants are at risk for increased oxidative stress due to necessary life-sustaining medical therapies while in the neonatal intensive care unit (NICU) [22]. Thus, understanding the different plasma carotenoid concentrations in different gestational age groups will enhance our understanding of neonatal nutrition and could guide future nutrition support interventions in preterm infants to optimize their nutritional status and ideally mimic intrauterine nutrient accrual. Therefore, the primary purpose of this study was to assess the maternal and umbilical cord plasma concentrations and the maternal-umbilical cord plasma ratios of the most prevalent six carotenoids in the US diet across five different groups of birth gestational age.

## 2. Materials and Methods

### 2.1. Participant Enrollment

The study was approved by the Institutional Review Board at the University of Nebraska Medical Center IRB (#112-15-EP, Omaha, NE, USA). Eligible mothers were screened and enrolled at time of delivery; mothers gave written consent for both herself and her infant prior to participation. Inclusion criteria consisted of mothers ≥19 years of age admitted to the Labor and Delivery Unit and delivered at least one live-born infant. Exclusion criteria included liver, kidney, or gastrointestinal disease in the mother or infant that would affect normal nutrient metabolism, inborn errors of metabolism, congenital abnormalities in the infant, or infants who were deemed wards of the state.

### 2.2. Biological Samples Collection

Maternal blood samples were collected during routine labs upon admission for delivery. Umbilical cord blood samples were collected from cord blood drawn from every infant at the time of delivery. All collected blood samples were immediately processed and frozen at −80 °C and protected from heat and light to preserve nutrient integrity.

### 2.3. Carotenoid Laboratory Analysis

Carotenoids analyzed included α-carotene, β-carotene, lycopene, combined lutein + zeaxanthin, and β-cryptoxanthin. The first 299 dyad samples were analyzed at the Nutritional Biomarker Lab at the Harvard T. H. Chan School of Public Health via HPLC [23] using NIST standards, as previously described by Thoene et al. [9]. The remaining 71 dyad samples were analyzed at the University of Nebraska Medical Center using LC-MS/MS. Plasma carotenoid levels were measured in 100 µL aliquots. The antioxidants and internal standards were added, followed by Folch extraction, separation of the organic phase, evaporated to dryness and reconstituted in ethanol and acetonitrile and placed in an Acquity UPLC system equipped with ANSI-compliant well plate holder coupled to a Thermo Scientific TSQ Quantis mass spectrometer equipped with an APCI source. Then 10 μL was injected onto the column (Phenomenex Luna Omega C18, 1.6 μm, 100 Å, 2.1 mm × 100 mm) using water (0.1% *v*/*v* acetic acid) (solvent A) and methanol (0.1% *v*/*v* acetic acid) (solvent B) as mobile phase. The total run time is 15 min at a flow rate of 500 μL/min. Each carotenoid was analyzed by selective reaction monitoring (SRM). Quantitation was achieved by using a cocktail of internal standards and the concentrations were normalized to the amount of sample and reported as mcg/L. Each batch of samples run included several replicates of a plasma pool sample set. Quality control at both labs was achieved through the use of NIST standards.

### 2.4. Dietary Intake and Birth Data Collection

The Harvard Willett Food Frequency Questionnaire was administered to all mothers at the time of delivery for quantification of dietary intake over the course of pregnancy [24]. Demographic and clinical information for both mother and infant was collected from the electronic medical record. Demographic variables included maternal age, race, pre-pregnancy body mass index (BMI) in kilograms/meters^2^ (kg/m^2^), smoking status, and sex of infant. Clinical variables included mode of delivery (vaginal versus cesarean), infant corrected gestational age (CGA) at birth, and birth weight (grams, g), birth length and head circumference (cm) with growth percentiles (based on 2006 WHO growth chart for term infants (≥37 weeks CGA [25]) and 2013 Fenton preterm growth chart (infants born < 37 weeks CGA [26])), maternal pregnancy complications including gestational diabetes and preeclampsia, and neonatal complications such as respiratory distress syndrome or NICU admission. Gestational age groups were defined as extremely preterm (<28 weeks CGA), very preterm (28 to <32 weeks CGA), moderately preterm (32 to <37 weeks CGA), early term (37 to <39 weeks CGA), and term (≥39 weeks CGA) [27,28].

### 2.5. Statistical Analysis

Descriptive statistics were evaluated for all variables and counts and proportions for categorical variables. The maternal-umbilical cord plasma ratio was calculated by dividing maternal plasma concentration by umbilical cord plasma concentration. Median of raw maternal and cord concentrations by CGA group and median of all ratios by CGA group are presented in the Results. The Kruskal-Wallis test was used to compare maternal-umbilical cord plasma ratios and maternal dietary intake between groups of birth CGA. Post-hoc pairwise comparisons were conducted to compare median values of continuous variables between gestational age groups. Multiple linear regression was used to predict changes in maternal-umbilical cord plasma ratios based on birth CGA group after adjusting for maternal smoking and pre-pregnancy BMI. Due to the non-normal distribution of maternal-cord nutrient levels, a log transformation was applied, which satisfied model assumptions. In pregnancies with multiple gestation, only infant A was included in analyses. IBM SPSS Statistics 26 software (IBM Corp., New York, NY, USA) was used for all statistical analyses. A *p*-value < 0.05 was considered statistically significant.

## 3. Results

### 3.1. Demographic and Birth Data

A total of 370 mother-infant dyads were included in this analysis. The median infant birth CGA was 39.3 weeks, and the median birthweight was 3374 g. There were 201 male infants (54.3%) and 169 female infants (45.7%) in the study. The majority of the maternal delivering population was white (*n* = 235, 63.5%), following by African American (*n* = 57, 15.4%), Hispanic (*n* = 37, 10.0%), Other/Unknown (*n* = 31, 8.4%), Asian or Pacific Islander (*n* = 9, 2.4%), and American Indian (*n* = 1, 0.3%). Demographic data by birth CGA group is presented in Table 1.

### 3.2. Maternal Carotenoid Intake

Maternal dietary intake data was available on 305 subjects, none of which were extremely preterm. The maternal dietary intake of α-carotene, β-carotene, lycopene, lutein + zeaxanthin was the same across the categories of birth CGA (*p* = 0.46, 0.13, 0.22, 0.11 respectively) (Table 1). Maternal dietary intake of β-cryptoxanthin, however, did vary with birth CGA (*p* = 0.01). More specifically, there were statistically significant differences in intake of β-cryptoxanthin between term and very preterm infants (0.1 vs. 0.3, *p* = 0.01), early term and very preterm infants (0.1 vs. 0.3, *p* = 0.001), and moderately preterm and very preterm infants (0.1 vs. 0.3, *p* = 0.03).

### 3.3. Carotenoid Concentrations and Maternal-Umbilical Cord Plasma Ratios

**Figure 1:**
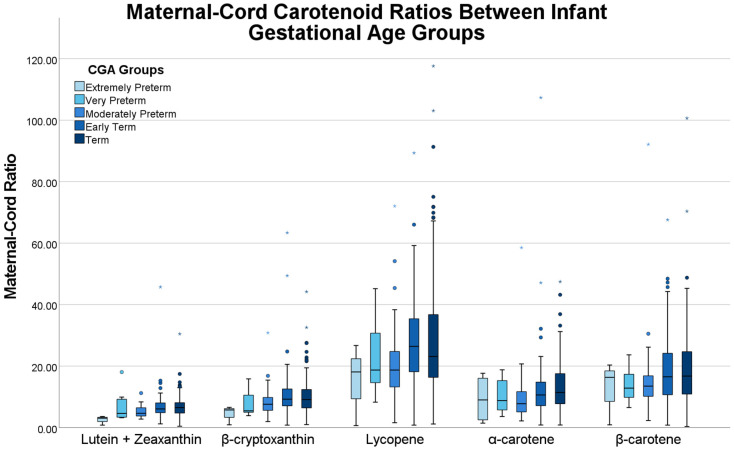
Maternal-umbilical cord plasma ratio of each carotenoid by birth corrected gestational age group.

The median, minimum, and maximum raw maternal and umbilical cord carotenoid concentrations by CGA group are presented in Table 2. In addition, the median of all individual maternal:cord ratios (see Methods) by birth CGA group are also listed in Table 2. Likewise, the maternal-umbilical cord plasma ratios are displayed in Figure 1. Across the five birth CGA groups, the maternal plasma concentrations varied significantly for all carotenoids (lutein + zeaxanthin, *p* < 0.001), β-cryptoxanthin, *p* < 0.001, lycopene, *p* = 0.005, and α-carotene, *p* = 0.007) with the exception of β-carotene which approached significance (*p* = 0.053). The umbilical cord plasma concentrations did not significantly vary across the birth CGA groups except β-cryptoxanthin (*p* = 0.008). However, the maternal-umbilical cord plasma ratio varied significantly between birth CGA groups for lutein + zeaxanthin (*p* = 0.008), β-cryptoxanthin (*p* = 0.027), and α-carotene (*p* = 0.030) and approached significance for lycopene (*p* = 0.056). Linear regression after adjusting for smoking status and maternal pre-pregnancy BMI did not yield statistically significant results.

## 4. Discussion

Data from this cohort demonstrates that maternal plasma carotenoid concentrations primarily increase with advancing gestational age groups. Maternal plasma carotenoid concentrations increased throughout gestation without a statistically significant change in dietary carotenoid intake. Though maternal concentrations increased, we observed less variability in newborn umbilical cord plasma concentrations, which is likely why maternal-umbilical cord plasma ratios increased with advancing birth CGA groups.

There are multiple considerations for these observed trends in maternal and umbilical cord plasma carotenoid concentrations and resulting maternal-umbilical cord plasma ratios. First, since maternal lipid levels increase throughout gestation, the increase observed in the maternal carotenoid plasma concentrations could be due to increased amounts of transporter lipoproteins in the plasma and thus more fat-soluble nutrients in the plasma are able to be quantified [15,16]. Likewise, pregnancy is a time of high exposure to oxidative stress, so maternal carotenoid concentrations could be lower at earlier gestational ages due to antioxidant consumption by the placenta or by the fetus at critical stages of development [11]. However, it is unknown how maternal tissue concentrations are affected by these higher carotenoid concentrations detected in plasma. In comparison with our observations, prior research has mixed results when comparing repeated carotenoid measurement throughout a full-term pregnancy. In example from a Peruvian cohort, Horton et al. reported higher mean maternal plasma concentrations in the third trimester of pregnancy (compared to trimester 1 or 2) for specific carotenoids such as α-carotene, β-carotene, and lycopene, but no differences were observed in others such as β-cryptoxanthin [29]. However, unique to our study is that we compared carotenoid concentrations between varying birth gestational ages as opposed to repeated measurement across full-term pregnancies. Though causal relationships cannot be determined, we question if maternal concentrations at earlier birth gestational ages are lower than expected compared to concentrations in a full-term pregnancy due to factors associated with increased oxidative stress and preterm delivery. Likewise, it is undetermined if low maternal carotenoid concentrations were a precipitating risk for preterm delivery, as past research has reported higher risk of preterm delivery in pregnant women with low plasma carotenoid concentrations [30].

Lastly, it must be recognized that there currently are no recommended reference ranges for maternal or infant plasma carotenoid concentrations. This is evidenced by a wide range of observed values in our study cohort, thus comparing concentrations within the different birth CGA groups is challenging. However, these results mimic our prior research where we observed a certain level of intrauterine retinol transfer to the fetus was maintained regardless of maternal plasma retinol status, leading to similar newborn retinol concentrations at time of birth [31]. That said, it is still undetermined if body tissue carotenoid concentrations are more variable than plasma concentrations at disparate birth sizes and gestational ages [31]. Therefore, more research is needed to investigate the mechanisms of intrauterine carotenoid transfer with the goal of identifying reference ranges for dietary intake and plasma concentrations for mothers and both term and preterm infants.

### 4.1. Implications for Clinical Practice

Preterm infants, especially their under-developed lungs and eyes, are highly susceptible to oxidative stress due to their low antioxidant concentrations, extensive metabolic activity, and increased production of free radicals [32]. This is of upmost importance as increased concentrations of carotenoids in preterm infants is associated with less retinopathy of prematurity, necrotizing enterocolitis, and bronchopulmonary dysplasia [32,33]. Even though there were few significant differences in umbilical cord plasma carotenoid concentrations between the varying birth CGA groups, there remains uncertainty about the concentrations in different body tissues, in particular the eyes and brain. For example, Vishwanathan et al. found lower lutein concentrations in brain tissue of preterm compared to term infants, demonstrating the low nutrition status of this population and the importance of adequate postnatal supplementation [34]. Receiving appropriate nutrition for adequate growth is critical for a preterm infant and provision of maternal breast milk is the ideal source of nutrition [35]. However, our observations of lower maternal-umbilical cord plasma carotenoid ratios in the preterm CGA groups – especially those born very or extremely preterm – warrants concern, as the quantity of carotenoids present in maternal plasma is lower than at later gestational ages. After birth, the carotenoid concentrations in breast fed infants is dependent upon the carotenoid concentration present in breast milk, which is affected by maternal dietary carotenoid intake and plasma carotenoid concentrations [36]. Additionally, prior research reports the colostrum of mothers who delivered preterm infants consisted of lower concentrations of all carotenoids when compared to the colostrum of mothers who delivered at term, with the exception of lutein which showed no difference between the groups [37]. Therefore, lower content of antioxidant carotenoids in maternal plasma and breastmilk around the time of birth and throughout NICU hospitalization for preterm infants may predispose these infants to poor outcomes and impeded development. As these nutrients are fat-soluble, cumulative intakes over time may have significant impact on associated clinical outcomes, such as with cumulative lutein intake in visual and cognitive functioning [38]. In further example from our work, we previously observed that maternal plasma carotenoid concentrations at the time of delivery are associated with greater anthropometric measurements in their newborns [39,40]. Though further research is necessary to understand the relationship between carotenoid concentrations in breast milk and tissue concentrations with neonatal growth outcomes, an association could have implications for the future nutrition management in preterm infants.

Consideration must also be given to infants born at early term. While this category of infants may receive standard newborn nutritional care, they may still be born with lower antioxidant nutrient stores that require appropriate postnatal supplementation. In support of this hypothesis, our data shows mothers delivering at early term have lower plasma carotenoid concentrations compared to those delivering just weeks later at full term. Though not statistically significant, most median maternal carotenoid concentrations were more than 15% higher at full term compared to early term deliveries. This again could impact carotenoid concentrations in the maternal breast milk these infants receive and resulting plasma concentrations could have additional implications for these infants’ growth and development.

Lastly, it is important to note that although breastmilk is considered the ideal source of nutrition for infants, many preterm infants receive heat-processed donor human milk, formula, human milk fortifiers, parenteral nutrition, or a combination of these – all of which may limit carotenoid provisions. In example, parenteral nutrition and some common North American infant formulas or human milk fortifiers contain either no carotenoids, a limited number, or low concentrations of carotenoids. For instance, we previously reported that pasteurized donor human milk consists of lower concentrations than maternal breast milk of all carotenoids [41]. Similarly, there may be differences in enteral absorption among preterm infants based on substrate, as those fed breast milk have been shown to have higher tissue concentrations of specific or total carotenoids than those fed formula [42,43,44]. As preterm infants are a high-risk population at baseline, future research is necessary to more definitively identify the most optimal dietary intakes and therapeutic plasma carotenoid concentrations. By understanding preterm infants’ baseline carotenoid status at birth, future interventions will allow us to observe how these plasma concentrations change over time while in the NICU. This can give greater insight into how we can better provide these carotenoids to infants in the NICU and help them combat both oxidative stress and neonatal disease. Similarly, by understanding the changes in carotenoid concentration over time in maternal breast milk, future nutritional interventions will aim to optimize maternal dietary intake to enhance breast milk carotenoid concentrations.

### 4.2. Limitations

One limitation of the study is that the vast majority of the dyads consisted mostly of early term and term healthy infants. There were substantially fewer extremely and very preterm infants enrolled in this study, which limited our ability to observe significant associations in the linear regression model, despite significant unadjusted relationships. In addition, there may be other variables we did not account for or evaluate that may impact plasma carotenoid concentrations and resulting maternal-umbilical cord plasma ratios, such as inflammatory biomarkers, pregnancy complications, or medication use. These variables should be particularly evaluated in preterm compared to term mother-infant dyads, as they may be more prevalent in the context of medical indications for preterm delivery. Likewise, these variables should be analyzed in future work in the context of a larger more heterogeneous sample size that includes a larger proportion of preterm infants.

## 5. Conclusions

Maternal plasma carotenoid concentrations increase with advancing birth gestational age while umbilical cord concentrations remain fairly similar, resulting in higher maternal-umbilical cord plasma ratios as birth gestational age increases. Further research is necessary to understand these mechanisms of action and to determine how these observed trends in earlier gestation compare to concentrations assessed throughout the course of a full-term pregnancy. Additionally, identifying recommended reference ranges for plasma carotenoid concentrations for pregnant women, preterm infants, and term infants is warranted to help determine the most optimal nutrition strategies in this perinatal population.

## Figures and Tables

**Table 1:** Demographic Data and Maternal Carotenoid Dietary Intake by each Gestational Age Group.

	Extremely Preterm <28 Weeks	Very Preterm 28 to <32 Weeks	Moderately Preterm 32 to <37 Weeks	Early Term 37 to <39 Weeks	Term ≥39 Weeks
Number of Infants	4 (1.1%)	11 (3.0%)	41 (11.1%)	75 (20.3%)	239 (64.4%)
Median CGA (weeks)	26.2 (23.8–27.8)	30.4 (29.1–31.3)	35.3 (34.0–36.3)	38.1 (37.6–38.6)	40.0 (39.4–40.7)
Median Birthweight (g)	1131	1485	2450	3062	3534
Median Pre-Gravid BMI (kg/m^2^)	N/A	24.5	29.5	25.9	26.0
**Delivery Mode, *n* (%)**					
Vaginal	3 (75.0%)	3 (27.3%)	24 (58.5%)	55 (73.3%)	180 (75.3%)
Cesarean Section	0 (0%)	8 (72.7%)	17 (41.4%)	20 (26.7%)	58 (24.3%)
Unknown	1	0	0	0	1
**Smoking Status, *n* (%)**					
Never Smoker	1 (25%)	8 (72.7%)	28 (68.3%)	55 (73.3%)	183 (76.6%)
Current/Former Smoker	2 (50%)	3 (27.3%)	13(31.7%)	19 (25.3%)	55 (23.0%)
Unknown	1 (25%)	0 (0%)	0 (0%)	1 (1.3%)	1 (0.4%)
**Maternal Dietary Intake**	*n =* 1	*n =* 7	*n =* 26	*n =* 62	*n =* 209
Lutein + zeaxanthin (mg/day)	N/A	3.9	3.0	2.0	2.4
β-cryptoxanthin* (mg/day)	N/A	0.3	0.1	0.1	0.1
Lycopene (mg/day)	N/A	6.0	6.5	4.2	4.6
α-carotene (mg/day)	N/A	0.3	0.6	0.5	0.5
β-carotene (mg/day)	N/A	5.0	5.8	4.4	5.0

* Indicates statistically significant differences between gestational age groups with *p* < 0.05.

**Table 2:** Median Raw Carotenoid Concentrations in Maternal Plasma (M), Umbilical Cord Plasma (UC), and Medians of Individual Maternal:Cord Plasma Ratios (R).

	Extremely Preterm (*n* = 3)			Very Preterm (*n* = 8)			Moderately Preterm (*n* = 29)			Early Term (*n* = 67)			Term (*n* = 192)			*p* ^a^
	M	UC	R	M	UC	R	M	UC	R	M	UC	R	M	UC	R	
Lutein + zeaxanthin (mcg/L)	**44.9** (43.8–181.5)	**49.8** (14.1–54.1)	**3.1:1**	**124.8** (84.4–252.1)	**21.8** (9.5–59.8)	**4.6:1**	**133.7** (45.1–443.8)	**30.7** (11.2–71.9)	**4.7:1**	**184.8** (53.1–495.3)	**27.8** (7.6–108.1)	**6.5:1**	**218.8** (25.7–605.5)	**32.2** (0.62–144.7)	**6.6:1**	**0.008**
β-cryptoxanthin (mcg/L)	**9.1** (8.5–143)	**9.0** (1.4–24.8)	**5.8:1**	**64.4** (24.1–180.1)	**12.9** (2.7–34.8)	**6.4:1**	**80.1** (8.74–285.7)	**9.7** (2.0–31.6)	**8.3:1**	**102.8** (16.8–307.2)	**11.2** (1.9–47.0)	**9.6:1**	**118.1** (16.6–538.6)	**13.4** (1.3–683.4)	**9.1:1**	**0.027**
Lycopene (mcg/L)	**403.8** (13.9–405.9)	**20.4** (15.1–22.4)	**18.1:1**	**355.1** (235.5–545.2)	**21.7** (8.8–46.4)	**18.7:1**	**455.8** (72.3–921.4)	**22.3** (1.6–139.7)	**20.0:1**	**468.4** (48.8–1521.7)	**16.5** (0.0–294.8)	**26.4:1**	**541.2** (25.8–1306.7)	**20.9** (5.0–161.9)	**23.1:1**	0.056
α-carotene (mcg/L)	**16.4** (3.0–68.4)	**3.9** (2.1–4.6)	**3.6:1**	**30.0** (13.1–102.1)	**2.5** (1.5–10.1)	**10.0:1**	**24.0** (4.6–388.4)	**3.7** (0.0–18.8)	**7.7:1**	**33.8** (6.8–1022.3)	**3.4** (0.0–70.3)	**10.6:1**	**42.9** (2.4–601.1)	**3.5** (0.0–939.4)	**11.2:1**	**0.030**
β-carotene (mcg/L)	**202.2** (11.1–220.8)	**11.9** (10.9–12.2)	**16.6:1**	**95.9** (40.4–202.5)	**7.5** (3.7–16.7)	**13.8:1**	**84.1** (23.1–1036.7)	**9.5** (1.6–62.0)	**13.4:1**	**147.0** (15.6–998.0)	**9.0** (0.0–61.3)	**16.7:1**	**187.7** (7.1–3003.1)	**10.0** (0.0–286.6)	**16.6:1**	0.201

^a^ Kruskal–Wallis test comparing differences in maternal-umbilical cord plasma ratios.

## Data Availability

Data available upon request to the corresponding author. This data is not publicly available due to ethical considerations regarding participant confidentiality. All of other data is contained within the article.
